# A Multi-Marker Genetic Association Test Based on the Rasch Model Applied to Alzheimer’s Disease

**DOI:** 10.1371/journal.pone.0138223

**Published:** 2015-09-17

**Authors:** Wenjia Wang, Jonas Mandel, Jan Bouaziz, Daniel Commenges, Serguei Nabirotchkine, Ilya Chumakov, Daniel Cohen, Mickaël Guedj, the Alzheimer’s Disease Neuroimaging Initiative

**Affiliations:** 1 Pharnext, Issy-les-Moulineaux, Ile de France, France; 2 Inserm U897, University of Bordeaux, Bordeaux, Aquitaine, France; University of Texas School of Public Health, UNITED STATES

## Abstract

Results from Genome-Wide Association Studies (GWAS) have shown that the genetic basis of complex traits often include many genetic variants with small to moderate effects whose identification remains a challenging problem. In this context multi-marker analysis at the gene and pathway level can complement traditional point-wise approaches that treat the genetic markers individually. In this paper we propose a novel statistical approach for multi-marker analysis based on the Rasch model. The method summarizes the categorical genotypes of SNPs by a generalized logistic function into a genetic score that can be used for association analysis. Through different sets of simulations, the false-positive rate and power of the proposed approach are compared to a set of existing methods, and shows good performances. The application of the Rasch model on Alzheimer’s Disease (AD) ADNI GWAS dataset also allows a coherent interpretation of the results. Our analysis supports the idea that *APOE* is a major susceptibility gene for AD. In the top genes selected by proposed method, several could be functionally linked to AD. In particular, a pathway analysis of these genes also highlights the metabolism of cholesterol, that is known to play a key role in AD pathogenesis. Interestingly, many of these top genes can be integrated in a hypothetic signalling network.

## Introduction

With the recent improvement of high-throughput genotyping technologies, the use of Genome-Wide Association Studies (GWAS) has become widespread in genetic research to identify significant associations between genetic markers such as Single Nucleotide Polymorphisms (SNPs) and complex phenotypes such as common diseases. GWAS generally yield results at the SNP-level, that are sets of SNPs associated with the disease. However, the vast majority of loci that have been identified for common diseases show modest effects and generally explain only a small part of the variance or heritability of the phenotype observed [1]. In a recent study of Body Mass Index (BMI), the markers associated explained only 0.84% of the variance, although it is considered that genetic factors should actually account for 40%-70% of the variance of BMI [2]. One explanation for the missing heritability is that the common analysis approach, assessing the effect of each SNP individually, is not well suited for the detection of small effects of multiple SNPs. Disease susceptibility is actually likely to depend on the cumulative effect of multiple variants in several genes interacting in functional pathways [3].

It is increasingly recognized that analyzing the combined association of multiple markers at the gene or pathway level may provide a complementary approach to the more common single SNP association approach, with several key benefits [4]. First it incorporates a priori biological knowledge in the analysis: as a matter of fact, in Genetics, the gene is often considered as the unit of interest since the analyses of the functional mechanisms of a disease are generally based on genes and their products such as RNA or proteins [5]. Determining the genes associated with the disease opens the door to a lot of additional research such as targeting genes of interests for candidate-gene studies or replicate association studies. Also, it allows the consideration of biological information, such as pathways or protein interactions, in the analysis of GWAS [6]. For instance, enrichment analysis such as performed by the method Gene Set Enrichment Analysis (GSEA) [7] aims to determine sets of genes involved in common biological processes or biological pathways. Such an analysis is possible through the use of functional information that is only available at the gene level. Second, as the number of genes or pathways is substantially smaller than the number of markers genotyped in GWAS, fewer hypotheses will be tested requiring less stringent multiple-testing correction [8]. Finally, by combining SNPs with modest associations, evidence of association at the gene or pathway level may emerge, even when the analysis of individual SNPs failed to identify any significant association.

In this context, the measure that summarizes the association between multiple SNPs and the trait of interest into a single statistic is a crucial step that raises several statistical issues. Among them, the number of SNPs considered and the impact of the possible Linkage Disequilibrium (LD) between them are often considered [4]. The most widely used approach is the minimum *p*-value of all the SNPs assigned to the set of SNPs, i.e. the *p*-value of the most significant SNP [9]. However it focuses on the most significant SNP only, rather than using the information provided by all the SNPs simultaneously which can be view as a limitation. In addition when applied directly, it has an inflated false-positive rate as it does not account for the two statistical issues described above [10]. In order to correct for both the number of SNPs and the LD, a phenotype permutation procedure can be used [11]. But permutations are time consuming, particularly if we want to reach a sufficient level of precision on *p*-values. Over the years, a number of alternatives have been proposed, such as the the Fisher’s statistic to combine *p*-values of association over a set of SNPs [12].

Here we propose an adaptation of the Rasch model as a novel statistical approach to evaluate the combined effect of multiple genetic variants. Named after Georg Rasch, the Rasch model is a mathematical framework initially proposed to analyze rating scales and evaluates a latent variable not measurable directly from a set of categorical items (eg, disability, cognition or quality of life). The Rasch model is increasingly used in many areas of application such as Psychometry, Social Sciences, Education, and Clinical Trials [13], but has yet to be applied to Genetics. We believe that the application of the Rasch model to association studies offers a solution to the joint analysis of multiple genetic markers. Through different sets of simulations, the false-positive rate and power of the proposed approach is compared to a set of existing methods. By way of illustration, we also apply it to the Alzheimer ADNI GWAS data.

## Methods

### Introduction to the Rasch model

Some variables can be measured directly (eg, height and weight); other variables are measured indirectly by how they manifest (eg, disability, cognitive function, quality of life). Therefore, we need a method to transform the manifestations of these “latent” variables into numbers that can be taken as measurements [14]. **Rating scales** are a means to measure latent variables by a set of items, each of which has two or more ordered response categories that are assigned sequential integer scores.

For the analysis of rating scales, the **Classical Test Theory** is usually applied, whereby the item scores are summed to give a total score. However, this simple and natural approach has two main limitations [13]. First, scoring the items with sequential integers implies equal differences at the item level (differences between each response category are assumed to be equal) and at the summed score level (a change of one point implies an equal change across the range of the scale, no matter which item is concerned by this change). Consequently, such ordinal scores cannot provide us with a stable frame of reference in terms of the distance between individuals on the ability scale. Second, when applying the Classical Test Theory, the latent trait of interest is estimated by a summed score which is actually difficult to match to each single item in order to know what an individual can actually perform: individuals with the same summed score may not be able to achieve the same item task. To establish a reliable rating scale, the information of the relative difficulties of items which is actually lost in the summed score must be taken into account.

As a main alternative to overcome theses limitations, the **Item Response Theory** assumes that the probability of a specified score of a person on an item is a function of the person’s ability and the item difficulty [15]:

$$\begin{array}{c} \mathrm{Pr}\left(X_{ni}=x\right)=f\left(\beta_{n},\tau_{ki}\right), \end{array}$$

where *X*_*ni*_ = *x* ∈ {0, 1, …, *m*_*i*_} is an integer random variable for item *i* where *m*_*i*_ is the maximum score, *β*_*n*_ corresponds to the ability parameter of person *n* and *τ*_*ki*_ corresponds to the difficulty to obtain the score *k* for the item *i*. When the person’s ability is high and the item difficulty is low, the probability of having a high score for that item increases.

The **Rasch model** constitutes a particular case of the Item Response Theory and can be viewed as applying a transformation to the total scores [16]. The Rasch transformation preserves the order of the raw scores, but the distance between individuals can be assessed, and not only the rank ordering. Second, both the item difficulty and person ability are defined on the same scale; if a person’s ability is known, we can predict how that person is likely to perform on an item. The Rasch model has several forms and extensions according to the data. The simplest form is the **dichotomous Rasch model** and corresponds to the situation where items have only two response categories (0 and 1). Specifically, the probability of a correct response is modeled as a logistic function of the difference between the person and item parameter:

$$\begin{array}{c} \mathrm{Pr}\left(X_{ni}=1\right)=\frac{\exp(\beta_{n}-\tau_{1i})}{1+\exp(\beta_{n}-\tau_{1i})}. \end{array}$$

It assumes that when the person’s ability equals the item difficulty, the probability of score 1 for item *i* is 0.5. The **polytomous Rasch model** is a generalization of the dichotomous Rasch model [17]. Here, we will precisely consider the **Partial Credit model** which allows different difficulty parameters for different items [14]:

$$\begin{array}{c} \mathrm{Pr}\left(X_{ni}=x\right)=\frac{\exp\sum_{k=0}^{x}\left(\beta_{n}-\tau_{ki}\right)}{\sum_{j=0}^{m_{i}}\exp\sum_{k=0}^{j}\left(\beta_{n}-\tau_{ki}\right)}. \end{array}$$

The Rasch model is based on four assumptions: 1) in the model there is only one latent variable of interest, which is the focus of the measurement and all items tap into this latent variable; 2) the total scores over an item or a person contains sufficient information for calculation of the parameters of the model; 3) for a person, the response to different items are independent; 4) the relationship between the probability of a given score to an item *i* and the latent trait is described by a logistic curve. Based on these assumptions, the item difficulty parameters (*τ*_*ki*_) can be estimated by Conditional Maximum Likelihood; then the person’s ability parameters (*β*_*n*_) can be estimated by Maximum Likelihood.

### Application of the Rasch model to multi-marker genetic association

The Rasch model is a measurement model that has potential application in any context where the objective is to measure a trait or ability through a process in which responses to items are scored with successive integers. When dealing with bi-allelic SNPs of possible alleles *a* and *A*, a set of SNPs can be considered as a set of items of possible categories 0 (= *aa*), 1 (= *aA* or *Aa*) or 2 (= *AA*) assuming an additive effect which is a reasonable hypothesis for complex traits, and analyzed with the polytomous Rasch model in order to summarize the information into one score. It corresponds to the person’s ability parameter defined previously. In summary, our appraoch takes the genotypes of a set of SNPs as entry and apply the Rasch model to calculate one **multi-marker Rasch genetic score** per subject.

Once this score is estimated for each subject, its association to a trait of interest can be assessed within classical statistical inference models according to the trait of interest (linear for quantitative traits, logistic for binary traits) with the possibility to adjust with covariates such as population stratification or gender.

### Implementation with R

Several softwares and R packages are available for Rasch model analysis such as ConQuest (https://shop.acer.edu.au/group/CON3), RUMM (www.rummlab.com.au), ltm (cran.r-project.org/package=ltm) and eRM (cran.r-project.org/package=eRm). Considering its flexibility and ease of integration to a pipeline of analysis, we choose to use the eRM R package.

The following short R script provides the functions used to obtain the multi-marker Rasch genetic score for each subject of a dataset of interest, where ‘Geno’ is a data matrix of genoptypes coded by 0, 1 and 2, with subjects in rows and markers in columns:

> library(eRM)
> rasch.model = PCM(Geno)
> score = person.parameter(rasch.model)$theta.table[, 1]

If ‘Trait’ is a binary trait disease coded by 1 for cases and 0 for controls, the association of the multi-marker Rasch genetic score to the disease can then simply be assessed with a logistic model:

> glm(Trait ∼ score, family = “binomial”)

If ‘Trait’ is a quantitative trait, the association of the multi-marker Rasch genetic score to the disease can then simply be assessed with a linear model:

> lm(Trait ∼ score)

### Simulations

The performances of our Rasch-based multi-marker genetic association test are first evaluated in term of **false-positive rate** and **power** based on simulations over three scenarios of dependence between SNPs and varying levels of association. For each scenario, we consider:

– a binary disease trait (500 cases and 500 controls) of prevalence *K*_*p*_ = 0.05.
– a set of 24 SNPs including 12 disease susceptibility loci (DSL) simulated with relative risks ranging from 1 (no association) to 2 (strong association).

This simulation framework detailed hereafter follows principles widely used previously [18–22].

#### Scenario 1: SNPs are independent

The simulation model for one SNP is based on the Wright’s model [23] applied to a bi-allelic marker with alleles *a* and *A* having the frequencies *p*_*a*_ and *p*_*A*_ = 1 − *p*_*a*_. *p*_0_, *p*_1_ and *p*_2_ are the frequencies of genotypes *aa*, *aA*/*Aa* and *AA* defined by the Hardy-Weinberg proportions:

$$\begin{array}{c} \left\{ \begin{array}{c} p_{0}=p_{a}^{2}+Fp_{a}\left(1-p_{a}\right)\\ p_{1}=2p_{a}\left(1-p_{a}\right)-Fp_{a}\left(1-p_{a}\right)\\ p_{2}={\left(1-p_{a}\right)}^{2}+Fp_{a}\left(1-p_{a}\right) \end{array}\right. \end{array}$$

where *F* is the consanguinity coefficient. This coefficient can indicate a deficit (*F* > 0) or conversely an excess (*F* < 0) of heterozygous. Here, we consider *F* = 0, so that the locus is under the Hardy-Weinberg equilibrium. We then want to compute the genotype frequencies of the SNP for cases and controls *p*_*Di*_ and *p*_*Hi*_ where *i* = 0, 1 or 2 using the disease prevalence *K*_*p*_, the penetrances *f*_0_, *f*_1_ and *f*_2_ of the genotypes and the mode of inheritance. The main modes of inheritance can be defined by considering the relative risks $RR_{i/0}=RR_{i}=\frac{f_{i}}{f_{0}}$, i = 1, 2. By assuming an additive mode of inheritance ($RR_{1}=\frac{RR_{2}+1}{2}$), and using *f*_0_ = *K*_*p*_/(*p*_0_ + *RR*_1_ × *p*_1_ + *RR*_2_ × *p*_2_), *f*_1_ = *RR*_1_ × *f*_0_, *f*_2_ = *RR*_2_ × *f*_0_ and the Bayes formulas, we can easily derive the desired frequencies:

$$\begin{array}{c} \left\{ \begin{array}{c} \left(p_{D_{0}},p_{D_{1}},p_{D_{2}}\right)=\left(\frac{f_{0}\times p_{0}}{K_{p}},\frac{f_{1}\times p_{1}}{K_{p}},\frac{f_{2}\times p_{2}}{K_{p}}\right)\\ \left(p_{H_{0}},p_{H_{1}},p_{H_{2}}\right)=\left(\frac{\left(1-f_{0}\right)\times p_{0}}{K_{p}},\frac{\left(1-f_{1}\right)\times p_{1}}{K_{p}},\frac{\left(1-f_{2}\right)\times p_{2}}{K_{p}}\right) \end{array}\right. \end{array}$$

The 24 SNPs are simulated independently according to this model, the 12 non-associated SNPs with a relative risk of 1 and the 12 DSLs with a relative risk ranging from 1 to 2.

#### Scenario 2: SNPs in moderate Linkage Disequilibrium

To account for SNPs in Linkage Disequilibrium (LD), our simulation model follows an approach based on the diplotype frequencies of real datasets. These frequencies are used as an empirical distribution of the range of possible diplotypes. First, 12 DSLs are simulated independently from the model described in **Scenario 1**. Then the remaining SNPs are completed based on a real dataset (here the chromosome 6 of the ADNI dataset described below) in order to generate one LD blocks of moderate magnitude (0.4–0.7) around each DSL. Simulating this way leads to genetic patterns similar to those found in real data and therefore allows us to finely control the level of LD between SNPs.

#### Scenario 3: SNPs in strong Linkage Disequilibrium

The simulation is the same as for **Scenario 2** with the difference that we consider SNPs in strong LD (0.8–1).

#### Monte-Carlo estimation of false-positive rate and power

For each scenario and each level of DSL relative risk, we ran *B* = 1000 simulations in order to provide accurate Monte-Carlo estimates of false-positive rate and power. For each simulation we obtain a *p*-value of association of the set of SNPs simulated by applying our Rasch-based multi-marker association test. The false-positive rate is estimated by Pr_*H*_0__(*p*-value ⩽ *α*) and the power is estimated by Pr_*H*_1__(*p*-value ⩽ *α*), with *α* the significance level usually set to 5%. Consequently in our simulations, by placing ourselves under the null hypothesis *H*_0_ of no association (*RR*_2_ = 1), then under the alternative hypothesis *H*_1_ of association (*RR*_2_ > 1), we can respectively estimate both false-positive rate and power of our method by considering the same quantity:

$$\begin{array}{c} \frac{♯(p{\text{-value}}_{i}⩽\alpha ,i=1,...,B)}{B}, \end{array}$$

where ♯() represents the number of *p*-values inferior or equal to *α*.

#### Comparison to existing methods

We compared the performances of our Rasch-based multi-marker association test to three existing methods:

– minP [9] is the simplest and most naive method. It considers the most significant *p*-value of the set of SNPs considered as the *p*-value of the set. This method is obviously biased since it does not take the multiple-testing and the dependence of tests into account. It is used here as a negative control and also because it is nevertheless the most widely used approach in practice.
– GATES [24] is a multi-marker association test using an extended Simes procedure to apply on each SNP. The *p*-values computed by a standard linear trend test of association on each SNP are combined with the control of correlation structure: significant *p*-values in high LD count less than significant *p*-values of independent SNPs.
– Fisher [12] is the well-known Fisher’s combination of *p*-values. For *m* SNPs, the multi-marker test statistic is given by $T=-2\sum_{i=1}^{m}\text{ln}(p_{i})$ which has a chi-square distribution with 2*m* degrees of freedom under the null hypothesis when the *m* tests are independent. An adjustment to dependent tests is also available and used here [25].
– SKAT [26] is SNP-set Kernel Association Test. It aggregates individual test score statistics of SNPs in a set and efficiently computes the set-level p-value. It performs multiple regression of a phenotype on all variants with Davies method while adjusting for covariants for counting account for population stratification and upweights rare variants.

### Application to the Alzheimer ADNI GWAS data

Alzheimer’s disease (AD) is the most common neurodegenerative disorder and affects more than 35 million people worldwide. It is characterized by brain atrophy reflecting neuronal and synaptic loss and the presence of amyloid plaques and neurofibrillary tangles, leading to a progressive deterioration of cognitive functions involving memory, reason, judgment and orientation [27]. AD pathogenic mechanisms are still unclear and the disease remains a condition without cure. According to age at onset, two main types of AD are differentiated: Early-Onset AD (EOAD, appears generally before the age of 65, less than 10% of the AD population and clear genetic determinants with mutations found in the *APP*, *PSEN1* and *PSEN2* genes) and Late-Onset AD (LOAD, more than 90% of the AD population, appears generally after the age of 65 and has a complex etiology based on genetic and environmental factors) [28].

In recent years, several Genome-Wide Association Studies (GWAS) were performed to detect genetic loci associated with LOAD [29–31]. These studies support the hypothesis that *APOE* is a major susceptibility gene for LOAD [32]. In addition to *APOE*, markers within several other genes gave replicated evidence of association with LOAD [33]. The identification of these genes improves our knowledge of AD. For instance, *CR1* has been demonstrated to be able to produce an AD up-regulated protein [34]. Although these new loci have been found, some problems ramain unsolved. First, to date none of these loci has proven accurate or sensitive enough to serve as biomarker. Second, the replication of results is a tedious task in GWAS. To push the boundaries of current knowledge on AD, further studies about GWAS and statistical models are still necessary.

By way of illustration, we applied our Rasch-based multi-marker association test to the genes of the Alzheimer’s Disease Neuroimaging Initiative (ADNI) database (adni.loni.usc.edu) [31]. The study population is made up of 359 cases and 226 controls, genotyped with an Illumina Human 610-Quad (= 620901 SNPs). A standard quality control process based on minor allele frequency, Hardy-Weinberg equilibrium, missingness and relatedness excluded 31 cases, 49 controls and 82071 SNPs [35]. The dataset was also reduced with a minimal loss of information by pruning with Plink (window size = 50 SNPs, shift = of 5 SNPs at each step and threshold correlation coefficient of 0.2) [36]. Missing genotypes were imputed with weighted k-Nearest-Neighbors method [37]. SNPs are considered attached to a gene if they are located within a distance of 20 kb around it. The curated dataset to analyze comprises 16514 genes. For each gene and each subject, a Rasch-based multi-marker genetic score is computed, and the association of this score to the disease is evaluated by a logistic regression model.

The top genes identified by the Rasch analysis were integrated into a hypothetic signalling network. Protein-protein interaction data and functional findings were extracted from QIAGEN’s Ingenuity Pathway Analysis (IPA, QIAGEN Redwood City, www.qiagen.com/ingenuity), manually analysed and supplemented by literature curation.

## Results

### Simulations

**Fig 1:**
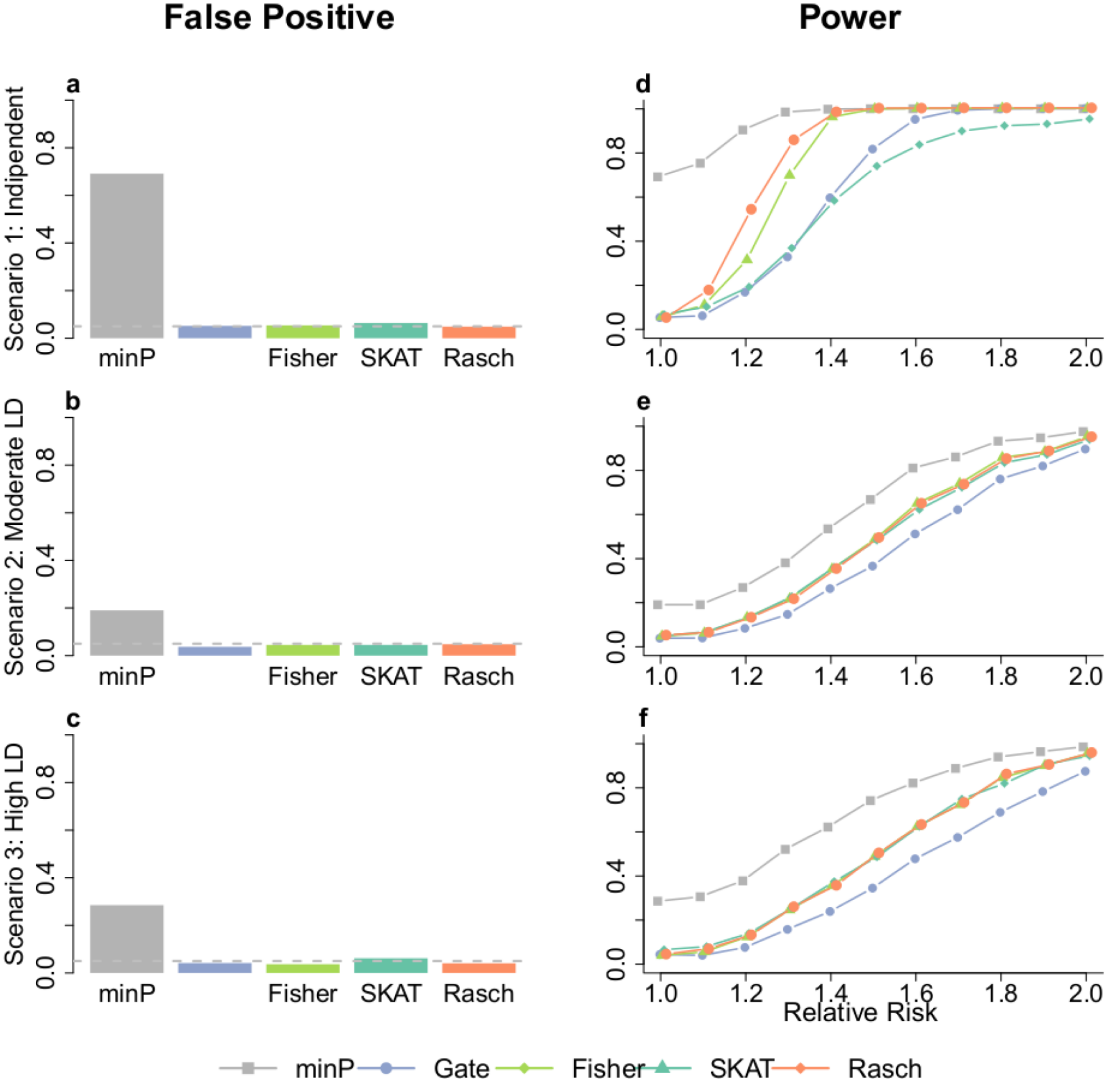
False-positive rate and power. Results of false-positive rate (a, b, c) and power (d, e, f) are given for the four methods (Rasch, minP, GATES, Fisher and SKAT) across the three scenarios.

False-positive rate and power for Rasch, minP, GATES, Fisher and SKAT across the three scenarios are given Fig 1. The first observation is that minP has a strongly inflated false-positive rate, far above the expected 5% level and decreasing with the level of LD (0.691 for **Scenario 1**, 0.285 for **Scenario 2** and 0.145 for **Scenario 3**). This observation was actually expected knowing the drawbacks of the minP method, and validates our simulations. On the other hand, Rasch, GATES, Fisher and SKAT have a correct control of the false-positive rate to 5% (Fig 1a,1b,1c) and a power that increases toward 100% with an increasing level of association to the disease (Fig 1d,1e,1f). In term of false-positive rate, it is worthy to mention that on **Scenario 3** (Fig 1c) Rasch is the closest to the 5% level (estimated to 0.043) whereas GATES and Fisher are more conservative (estimated to 0.039 and 0.034 respectively) and SKAT is more inflated. In term of power, Rasch has the best performances on independent SNPs followed by Fisher (**Scenario 1**, Fig 1d). Both methods have similar good performances when applied on SNPs with moderate and strong LD (**Scenarios 2,3**, Fig 1e,1f). The performance of GATES is better compared to SKAT on independent SNPs but is limited on LD block simulation. (Fig 1d,1e,1f).

### Application to the Alzheimer ADNI GWAS data

**Fig 2:**
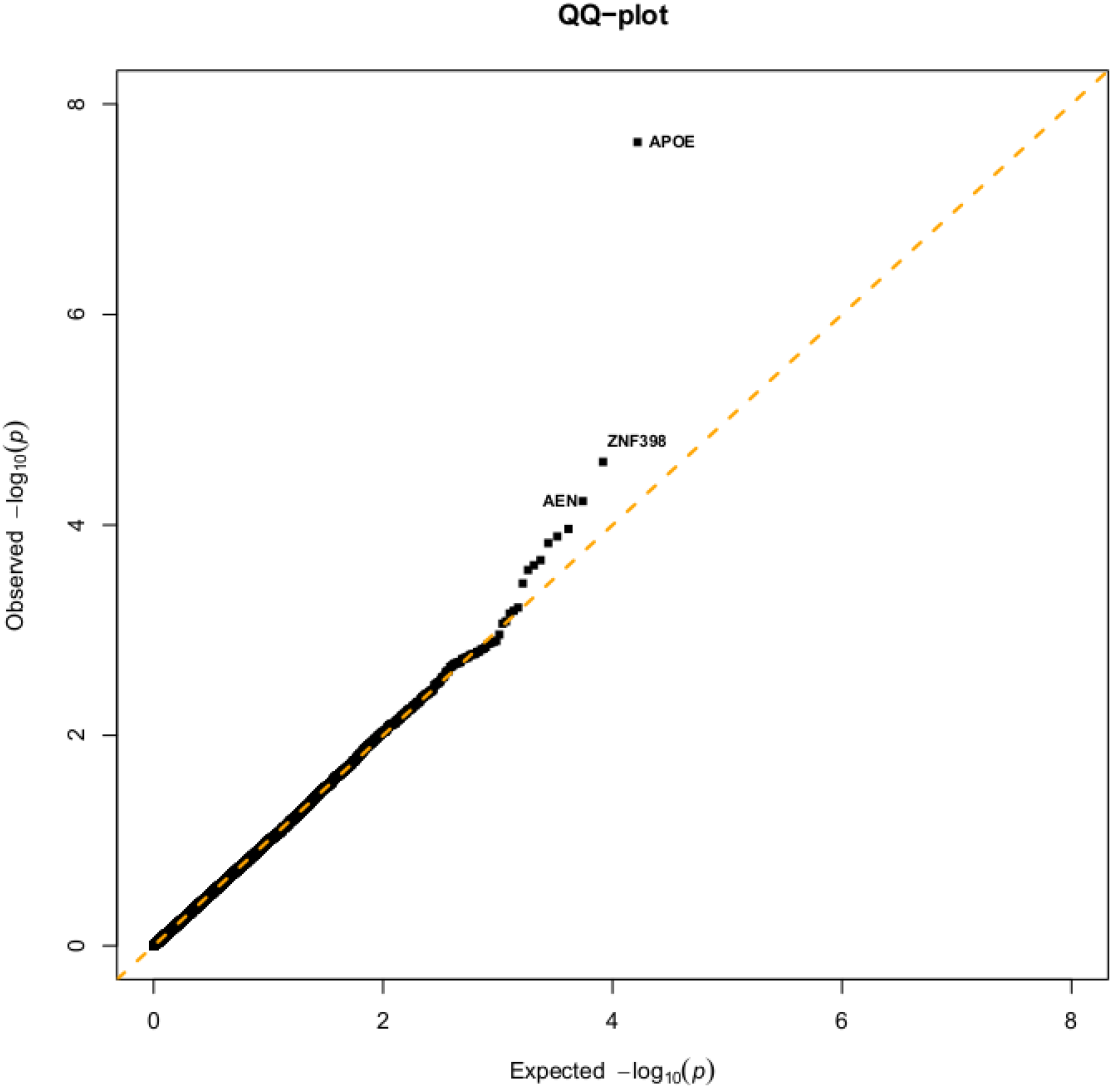
QQ plots. resulting from the application of the Rasch-based multi-marker association test to the genes of the Alzheimer ADNI GWAS dataset.

The association of 16514 genes to the Alzheimer’s disease (AD) was analyzed with our Rasch-based multi-marker association test. Standard QQ plots is given Fig 2 and the 20 top genes are detailed in Table 1. First, our analysis support the hypothesis that *APOE* on chromosome 19 is a major susceptibility gene for AD (*p* = 2.30*e* ^−8^). It is well-known that its *ɛ*4 allele has been associated with an increased risk of developing Alzheimer’s disease [38]. This result was expected and can be considered as a validation of our approach. Two other genes also markedly deviate from the QQ-line (Fig 2): *ZNF398* (*p* = 9.71*e* ^−6^) and *AEN* (*p* = 1.27*e* ^−5^). *AEN* encodes an enhancing apoptosis nuclease, a process that takes part to the neuronal loss observed in AD. We unfortunately did not find any indication about the possible functional implication of *ZNF398* in AD.

**Table 1:** Annotation of the 20 top genes resulting from the application of the Rasch-based multi-marker association test to the genes of the Alzheimer ADNI GWAS dataset.

Chromosome	Gene symbol	*p*-value	Full name	Location	Function
19	APOE	2.30e-08	Apolipoprotein E	Extracellular space	Transporter
7	ZNF398	9.71e-06	Zinc finger protein 398	Nucleus	Transcription regulator
15	AEN	1.28e-05	Apoptosis enhancing nuclease	Nucleus	Enzyme
7	ZNF425	1.37e-04	Zinc finger protein 425	Other	Other
5	ADAMTS12	1.59e-04	ADAM metallopeptidase with thrombospondin	Extracellular space	Peptidase
1	PSMA5	2.41e-04	Proteasome (prosome, macropain) subunit	Cytoplasm	Peptidase
13	FAM124A	2.85e-04	Family with sequence similarity 124A	Other	Other
9	FXN	4.29e-04	Frataxin	Cytoplasm	Kinase
11	NTM	4.80e-04	Neurotrimin	Plasma Membrane	Other
5	LARP1	5.01e-04	La ribonucleoprotein domain family	Cytoplasm	Translation regulator
1	WDTC1	5.39e-04	WD and tetratricopeptide repeats 1	Other	Other
11	EPS8L2	5.94e-04	EPS8-like 2	Other	Other
3	KBTBD12	6.27e-04	Kelch repeat and BTB domain containing 12	Other	Other
7	FAM188B	6.36e-04	Family with sequence similarity 188	Other	Other
17	OR3A3	6.94e-04	Olfactory receptor, family 3	Plasma membrane	G-protein coupled receptor
2	BZW1	7.17e-04	Basic leucine zipper and W2 domains 1	Cytoplasm	Translation regulator
23	TMEM187	7.62e-04	Transmembrane protein 187	Cytoplasm	Other
15	SEMA7A	7.87e-04	Semaphorin 7A, GPI membrane anchor	Plasma membrane	Transmembrane receptor
7	VKORC1L1	8.23e-04	Vitamin K epoxide reductase complex	Cytoplasm	Enzyme
19	COL5A3	9.80e-04	Collagen, type V, alpha 3	Extracellular space	Other

As we noticed a slight deviation from the QQ-line at 10^−3^ (Fig 2), we also investigated the other 17 top genes. Several of them could be functionally linked to AD:

– *PSMA5* is a proteasome subunit involved in the apoptosis process that takes part in the neuronal loss observed in AD [39]. *PSMA5* was also found to interact directly with the AD associated *PSEN1* gene [40].
– *FXN* encodes the frataxin mitochondrial protein which functions in regulating mitochondrial iron transport and respiration. Frataxin deficiency leads to mitochondrial dysfunction and oxidative damage that are at the origin of numerous neurodegenerative diseases like Friedreich ataxia, Parkinson and AD [41]. Interestingly, another top gene *VKORC1L1* is also involved in regulation of oxidative stress and mediates vitamin K-dependent intracellular antioxidant function [42]. Remarkably, blood level of vitamin K in *APOE4* carriers is lower than in persons with other *APOE* genotypes implying hypothetical link of vitamin K deficiency to pathogenesis of AD [43, 44].
– Alzheimer’s disease is sometimes named ‘type 3 diabetes’ due to twice more frequent occurrence in diabetic patients [45, 46]. Two top genes from our list (*COL5A3* and *WDTC1*) were identified as potent modulators of insulin signalling [47, 48]. Noteworthy, vitamin K-dependent modification of osteocalcin was also shown to affect glucose homeostasis [49].
– *NTM* encodes a neural cell adhesion molecule that modulates neurite outgrowth and adhesion via a homophilic mechanism [50]. Some data indicates that *NTM* might directly bind to amyloid beta [51]. It has been associated to intelligence in a family-based association study [52] and lies at locus 11q25 which has been associated with AD [53]
– *SEMA7A* belongs to the semaphorins family involved in neuronal processes. Semaphorins and their downstream signaling components regulate synaptic physiology and neuronal excitability in the mature hippocampus, and these proteins are also implicated in a number of developmental, psychiatric, and neurodegenerative disorders [54]. Remarkably, *SEMA7A* not only enhances axon growth via beta1-integrin, but equally processes immune-modulatory activity and regulates endothelial functions [55, 56]. As well, another top gene (*ADAMTS12*) is also implicated in control of immune responses and angiogenesis, deregulated in course of Alzheimer’s disease [57, 58].
– Finally, *LARP1* protein associates with the mTOR complex 1 (mTORC1) regulating global protein synthesis. Functional importance of mTOR signalling has been experimentally confirmed in Alzheimer’s disease, and therapeutic targeting of this signalling module is considered as a promising strategy for developing neuro-protective treatments [59–61].

**Fig 3:**
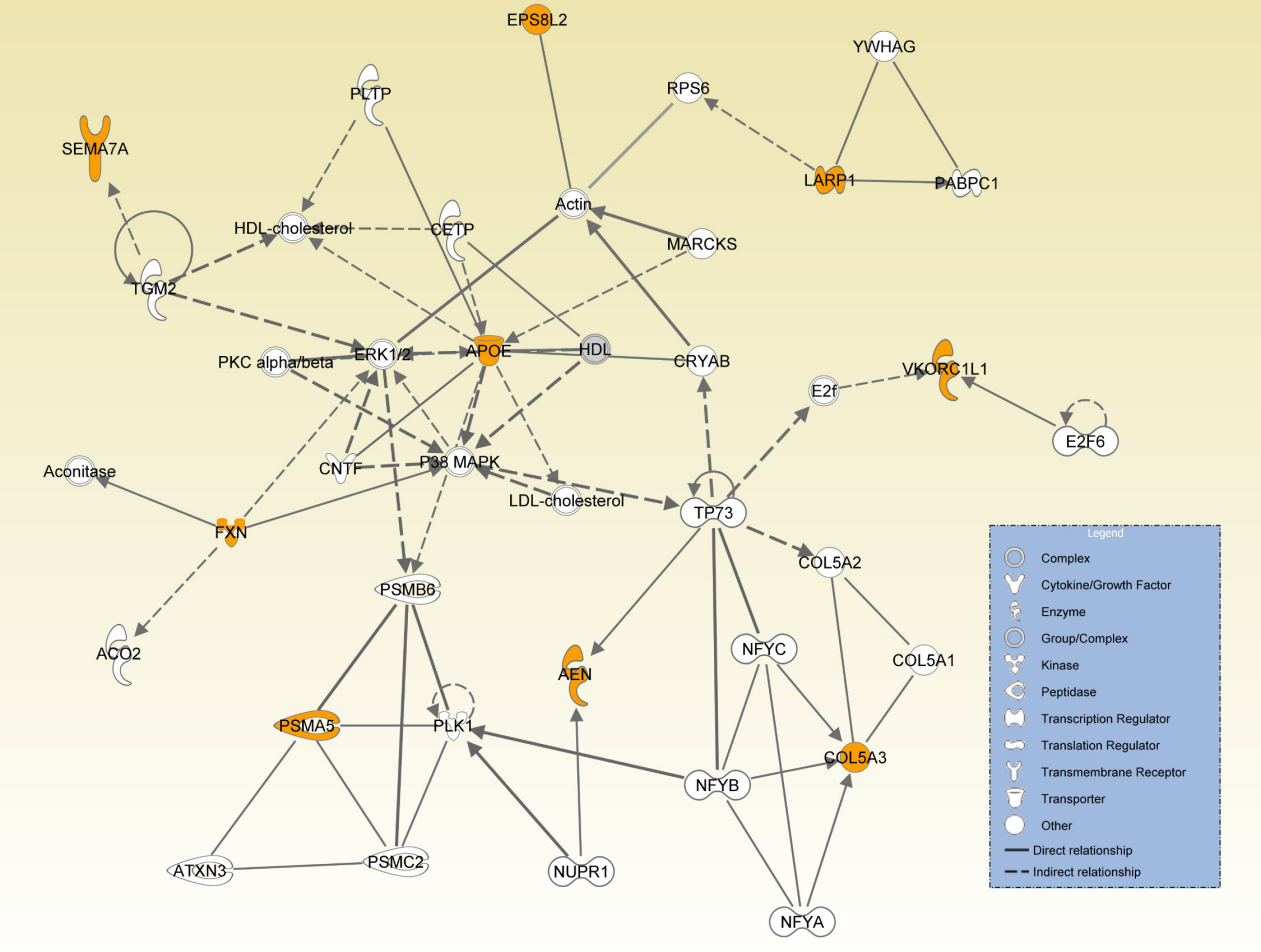
Ingenuity pathway analysis. resulting from the 20 top genes given in Table 1 (top genes are in orange).

We also performed a formalized network analysis based on these top genes with the Ingenuity Pathway Analysis. The resulting network is given Fig 3 and seems to highlight the metabolism of cholesterol that plays a key role in AD pathogenesis [62–64]. Nine of the 20 top genes are connected in this network (in orange). Remarkably, most of them can be functionally linked to AD. For example, integrin *ITGB1* mediates effect of *SEMA7A* on axon growth [65]. The integrins are modulated by *CASR* gene that forms a functional complex with metabotropic glutamate receptor *GRM5* [66, 67]. It was recently shown that *GRM5* is a co-receptor for cytotoxic A*β* oligomers bound to prion *PRNP* protein [68].

## Discussion

With the recent improvement of high-throughput genotyping technologies, the use of Genome-Wide Association Studies has become widespread in genetic research. However, the high dimension of the genetic data, the simultaneous testing of many markers and the necessity to account for the complex genetic structure of human populations are, among others, tricky issues that have raised doubts about the relevance of these studies’ findings. The development of methods in Statistical Genetics is therefore very important to ensure that such studies are correctly conducted and to provide a proper interpretation of their findings, and this research has involved scientists from many disciplines. In this context, applying the Rasch model initially developed for psychometric data to the analysis of genetic data can be viewed as a new link between two areas of research that was not obvious before. Our novel statistical approach may be useful to complement at the gene or pathway level, the findings of significant associations made at the single SNP level.

Based on simulations, it showed in different situations good performances in terms of false-positive rate and power compared to other popular methods (minP, GATES, Fisher and SKAT). We noticed that the benefits of Rasch in terms of power were more important when applied to independent SNPs which is coherent with one of the assumptions of the model that the response to different items are independent. As this loss of power is observed for all the methods when the level of dependence between the SNPs (Linkage Disequilibrium) increases, a Rasch model taking dependence into account could be of interest and further increase the power of the method.

**Fig 4:**
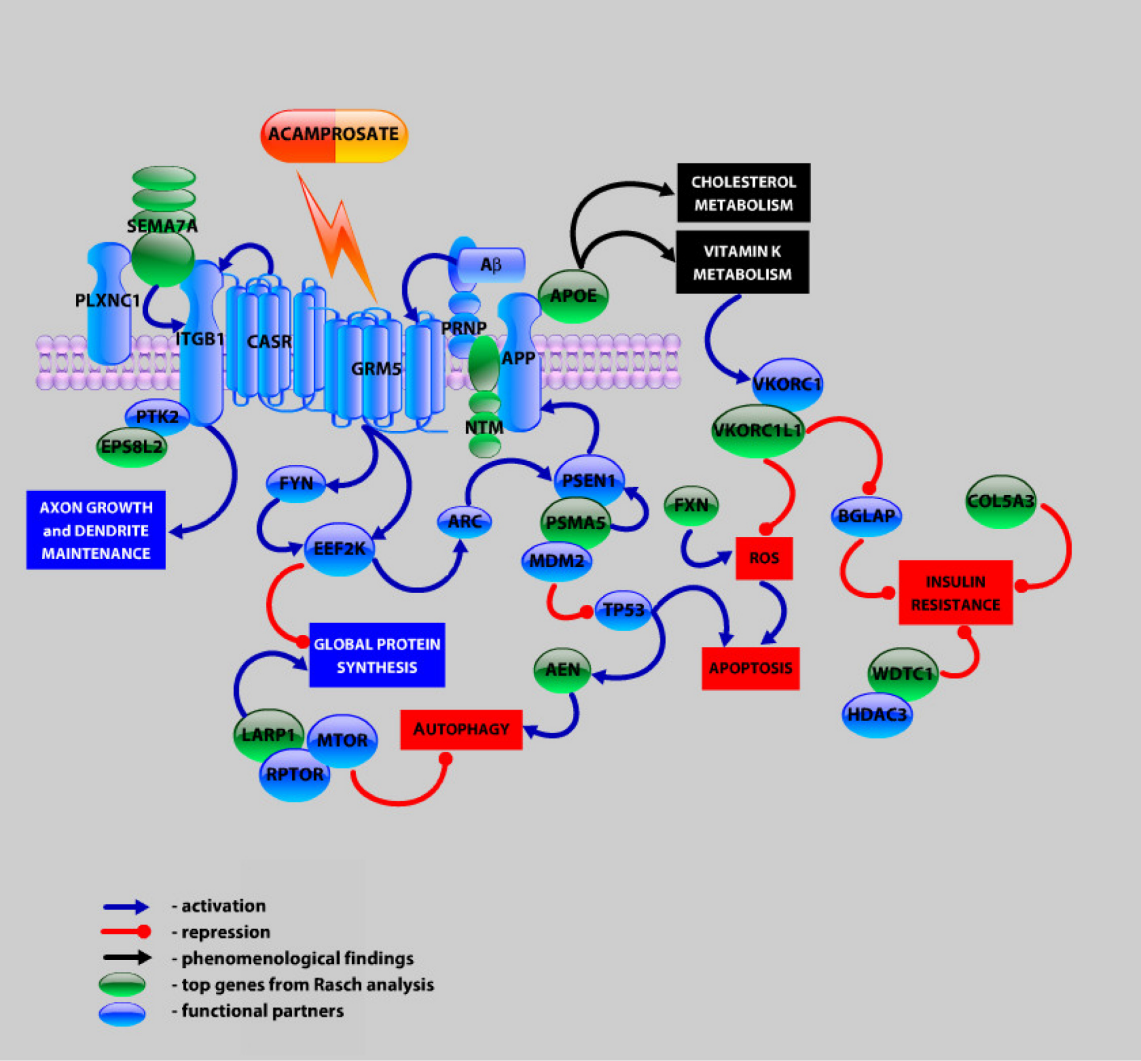
Hypothetic signalling network integrating top genes identified through the Rasch analysis. Gene abbreviations: *PLXNC1*—plexin C1 receptor for semaphorins; *PTK2*—*FAK* kinase implicated in integrin signalling; *FYN*—src family tyrosine kinase, downstream target of *GRM5* receptor; *EEF2K*—eukaryotic elongation factor-2 kinase, activated by *GRM5* receptor, regulates global protein synthesis; *MDM2*—negative modulator of *TP53* tumour suppression gene; *RPTOR*—regulatory protein associated with *MTORC1* complex; *HDAC3*—histone deacetylase; *ARC*—activity-regulated cytoskeleton-associated protein.

The application of the Rasch model to the genes of the Alzheimer ADNI GWAS data allowed a coherent interpretation of the data. Our analysis supports that *APOE* is a major susceptibility gene for AD. In the other top genes, several of them (*AEN*, *ADAMTS12*, *PSMA5*, *FXN*, *NTM*, *LARP1*, *WDTC1*, *SEMA7A*, *VKORC1L1*, *COL5A3*) can be functionally linked to Alzheimer’s disease. A pathway analysis of these genes also highlights the metabolism of cholesterol, that is known to play a key role in AD pathogenesis. All these elements can be integrated in a hypothetic signalling network based on known protein-protein, functional and phenomenological interactions (Fig 4). Interestingly, this network could be potentially targeted by acamprosate, a drug that was first approved in 1989 and since then has been widely used to treat alcohol-dependence [69]. In combination with baclofen, acamprosate has recently been shown to be effective over a range of preclinical AD models [70], and has demonstrated promising results in phase 2a clinical trial for AD [71].

Through this study, we encountered three limitations for the application of the Rasch model. First, it works on complete data without missing values. However missing values are a common problem in most scientific research domains as they can arise from different sources such as mishandling of samples, low signal-to-noise ratio, measurement error, non-response or deleted aberrant value. Consequently the application of the Rasch model requires preliminary imputation of missing values. This imputation is a general and separate scientific topic that has been thoroughly discussed to date [72–76]. Second, in some particular cases the estimation of the Rasch model with the eRM R package does not converge and consequently does not provide any results. It happened for instance to 9 genes over the 16514 genes analyzed in the ADNI GWAS data and the reasons of that problem were not clear to us. Finally applying a Rasch model necessitates accessing individual level genetic data. But often, only summary statistics are available for published GWAS. This is a real limitation for most of the existing multi-marker methods in order to correctly account for gene size and LD, although some authors have found a solution in using the genotype data from a reference panel such as the 1000 Genomes or the HapMap projects [77–79] which is not applicable here.

The application of the Rasch model also opens two opportunities that were not yet considered here. The analysis of multiple markers is not limited to the gene level, and the Rasch-based multi-marker genetic association test could also be applied to the analysis whole pathways. In addition, this genetic score could also be used as a predictor of the disease for the supervised classification of cases versus controls. The Rasch model is also suitable to the inclusion of rare variants, as most rare variants analyses focus on gene level test by collapsing the effects of all rare SNPs in a gene into a single test of association [4]. These applications deserve further investigation.

From a broader point of view, given the urgent need to understand how the thousands of loci that have been identified in genome-wide association studies contribute to the genetic basis complex traits, the application of multi-marker methods at the gene or pathway level becomes an increasingly important approach for secondary analysis of GWAS data [80–82]. Main recognized benefits include the incorporation of biological knowledge, the reduction in multiple-testing and the consideration of SNPs with modest effects. But this type of analysis has also clear limitations [4]. For instance determining whether a particular SNP is part of, or regulates a gene is a thorny problem. In addition, by focusing on SNPs that can be assigned to genes, analyzing GWAS data at the gene level also misses many disease associated SNPs that cannot be linked to genes (such as SNPs in gene deserts for instance). In that case, the delimitation of genomic regions made of contiguous SNPs and associated as a whole, should also complement our understanding of the genetic of complex traits [20].

## References

[1] Maher B (2008) Personal genomes: The case of the missing heritability. Nature 456: 18–21. 10.1038/456018a 18987709

[2] Willer CJ, Speliotes EK, Loos RJ, Li S, Lindgren CM, et al (2008) Six new loci associated with body mass index highlight a neuronal influence on body weight regulation. Nature genetics 41: 25–34. 10.1038/ng.287 19079261 PMC2695662

[3] Lehne B, Lewis CM, Schlitt T (2011) From snps to genes: disease association at the gene level. PloS one 6: e20133 10.1371/journal.pone.0020133 21738570 PMC3128073

[4] Fridley BL, Biernacka JM (2011) Gene set analysis of snp data: benefits, challenges, and future directions. European Journal of Human Genetics 19: 837–843. 10.1038/ejhg.2011.57 21487444 PMC3172936

[5] Jorgenson E, Witte JS (2006) A gene-centric approach to genome-wide association studies. Nature Reviews Genetics 7: 885–891. 10.1038/nrg1962 17047687

[6] Neale BM, Sham PC (2004) The future of association studies: gene-based analysis and replication. The American Journal of Human Genetics 75: 353–362. 10.1086/423901 15272419 PMC1182015

[7] Subramanian A, Tamayo P, Mootha VK, Mukherjee S, Ebert BL, et al (2005) Gene set enrichment analysis: a knowledge-based approach for interpreting genome-wide expression profiles. Proceedings of the National Academy of Sciences of the United States of America 102: 15545–15550. 10.1073/pnas.0506580102 16199517 PMC1239896

[8] Bouaziz M, Jeanmougin M, Guedj M (2012) Multiple testing in large-scale genetic studies In: Data Production and Analysis in Population Genomics, Springer pp. 213–233. 10.1007/978-1-61779-870-2_13 22665284

[9] Torkamani A, Topol EJ, Schork NJ (2008) Pathway analysis of seven common diseases assessed by genome-wide association. Genomics 92: 265–272. 10.1016/j.ygeno.2008.07.011 18722519 PMC2602835

[10] Hong MG, Pawitan Y, Magnusson PK, Prince JA (2009) Strategies and issues in the detection of pathway enrichment in genome-wide association studies. Human Genetics 126: 289–301. 10.1007/s00439-009-0676-z 19408013 PMC2865249

[11] Evangelou M, Rendon A, Ouwehand WH, Wernisch L, Dudbridge F (2012) Comparison of methods for competitive tests of pathway analysis. PloS one 7: e41018 10.1371/journal.pone.0041018 22859961 PMC3409204

[12] Whitlock M (2005) Combining probability from independent tests: the weighted z-method is superior to fisher’s approach. Journal of evolutionary biology 18: 1368–1373. 10.1111/j.1420-9101.2005.00917.x 16135132

[13] Hobart JC, Cano SJ, Zajicek JP, Thompson AJ (2007) Rating scales as outcome measures for clinical trials in neurology: problems, solutions, and recommendations. The Lancet Neurology 6: 1094–1105. 10.1016/S1474-4422(07)70290-9 18031706

[14] Masters GN (1982) A rasch model for partial credit scoring. Psychometrika 47: 149–174. 10.1007/BF02296272

[15] Woods A, Baker R (1985) Item response theory. Language Testing 2: 117–140. 10.1177/026553228500200202

[16] Rasch G (1960) Studies in mathematical psychology: I. probabilistic models for some intelligence and attainment tests.

[17] Andrich D, Sheridan B, Lyne A, Luo G (2000) Rumm: A windows-based item analysis program employing rasch unidimensional measurement models. Perth, Australia: Murdoch University.

[18] Bouaziz M, Ambroise C, Gued M (2011) Accounting for population stratification in practice: A comparison of the main strategies dedicated to genome-wide association studies. PloS one 6(12): e28845 10.1371/journal.pone.0028845 22216125 PMC3244428

[19] Chapman JM, Cooper JD, Todd JA, Clayton DG (2003) Detecting disease associations due to linkage disequilibrium using haplotype tags: a class of tests and the determinants of statistical power. Human heredity 56: 18–31. 10.1159/000073729 14614235

[20] Guedj M, Robelin D, Hoebeke M, Lamarine M, Wojcik J, et al (2006) Detecting local high-scoring segments: A first-stage approach for genome-wide association studies. Statistical applications in genetics and molecular biology 5 10.2202/1544-6115.1192 17049033

[21] Wang T, Elston RC (2007) Improved power by use of a weighted score test for linkage disequilibrium mapping. The american journal of human genetics 80: 353–360. 10.1086/511312 17236140 PMC1785334

[22] Pan W (2009) Asymptotic tests of association with multiple snps in linkage disequilibrium. Genetic epidemiology 33: 497–507. 10.1002/gepi.20402 19170135 PMC2732754

[23] Wright BD, Masters GN (1982) Rating Scale Analysis. Chicago: MESA Press.

[24] Li MX, Gui HS, Kwan JS, Sham PC (2011) Gates: a rapid and powerful gene-based association test using extended simes procedure. The American Journal of Human Genetics 88: 283–293. 10.1016/j.ajhg.2011.01.019 21397060 PMC3059433

[25] Moskvina V, O’Dushlaine C, Purcell S, Craddock N, Holmans P, et al (2011) Evaluation of an approximation method for assessment of overall significance of multiple-dependent tests in a genomewide association study. Genetic epidemiology 35: 861–866. 10.1002/gepi.20636 22006681 PMC3268180

[26] Wu MC, Lee S, Cai T, Li Y, Boehnke M, et al (2011) Rare-variant association testing for sequencing data with the sequence kernel association test. Am J Hum Genet 89: 82–93. 10.1016/j.ajhg.2011.05.029 21737059 PMC3135811

[27] Salek RM, Xia J, Innes A, Sweatman BC, Adalbert R, et al (2010) A metabolomic study of the crnd8 transgenic mouse model of alzheimer’s disease. Neurochemistry international 56: 937–947. 10.1016/j.neuint.2010.04.001 20398713

[28] Rogaeva E (2002) The solved and unsolved mysteries of the genetics of early-onset alzheimer’s disease. Neuromolecular medicine 2: 1–10. 10.1385/NMM:2:1:01 12230301

[29] Harold D, Abraham R, Hollingworth P, Sims R, Gerrish A, et al (2009) Genome-wide association study identifies variants at clu and picalm associated with alzheimer’s disease. Nature genetics 41: 1088–1093. 10.1038/ng.440 19734902 PMC2845877

[30] Seshadri S, Fitzpatrick AL, Ikram MA, DeStefano AL, Gudnason V, et al (2010) Genome-wide analysis of genetic loci associated with alzheimer disease. Jama 303: 1832–1840. 10.1001/jama.2010.574 20460622 PMC2989531

[31] Potkin SG, Guffanti G, Lakatos A, Turner JA, Kruggel F, et al (2009) Hippocampal atrophy as a quantitative trait in a genome-wide association study identifying novel susceptibility genes for alzheimer’s disease. PloS one 4: e6501 10.1371/journal.pone.0006501 19668339 PMC2719581

[32] Coon KD, Myers AJ, Craig DW, Webster JA, Pearson JV, et al (2007) A high-density whole-genome association study reveals that apoe is the major susceptibility gene for sporadic late-onset alzheimer’s disease. Journal of Clinical Psychiatry 68: 613 10.4088/JCP.v68n0419 17474819

[33] Lee JH, Cheng R, Barral S, Reitz C, Medrano M, et al (2011) Identification of novel loci for alzheimer disease and replication of clu, picalm, and bin1 in caribbean hispanic individuals. Archives of Neurology 68: 320–328. 10.1001/archneurol.2010.292 21059989 PMC3268783

[34] Corneveaux JJ, Myers AJ, Allen AN, Pruzin JJ, Ramirez M, et al. (2010) Association of cr1, clu, and picalm with alzheimer’s disease in a cohort of clinically characterized and neuropathologically verified individuals. Human molecular genetics: ddq221. 10.1093/hmg/ddq221 PMC2908469 20534741

[35] Weale ME (2010) Quality control for genome-wide association studies In: Genetic Variation, Springer pp. 341–372. 10.1007/978-1-60327-367-1_19 20238091

[36] Purcell S, Neale B, Todd-Brown K, Thomas L, Ferreira MA, et al (2007) Plink: a tool set for whole-genome association and population-based linkage analyses. The American Journal of Human Genetics 81: 559–575. 10.1086/519795 17701901 PMC1950838

[37] Schwender H (2012) Imputing missing genotypes with weighted k nearest neighbors. Journal of Toxicology and Environmental Health, Part A 75: 438–446. 10.1080/15287394.2012.674910 22686303

[38] Corder E, Saunders A, Strittmatter W, Schmechel D, Gaskell P, et al (1993) Gene dose of apolipoprotein e type 4 allele and the risk of alzheimer’s disease in late onset families. Science 261: 921–923. 10.1126/science.8346443 8346443

[39] Wu Y, Deng Y, Zhang S, Luo Y, Cai F, et al (2015) Amyloid-*β* precursor protein facilitates the regulator of calcineurin 1-mediated apoptosis by downregulating proteasome subunit *α* type-5 and proteasome subunit *β* type-7. Neurobiology of aging 36: 169–177. 10.1016/j.neurobiolaging.2014.07.029 25194880

[40] Van Gassen G, De Jonghe C, Pype S, Van Criekinge W, Julliams A, et al (1999) Alzheimer’s disease associated presenilin 1 interacts with hc5 and zeta, subunits of the catalytic 20s proteasome. Neurobiology of disease 6: 376–391. 10.1006/nbdi.1999.0265 10527805

[41] Gomes CM, Santos R (2013) Neurodegeneration in friedreich’s ataxia: From defective frataxin to oxidative stress. Oxidative medicine and cellular longevity 2013 10.1155/2013/487534 PMC3725840 23936609

[42] Westhofen P, Watzka M, Marinova M, Hass M, Kirfel G, et al (2011) Human vitamin k 2, 3-epoxide reductase complex subunit 1-like 1 (vkorc1l1) mediates vitamin k-dependent intracellular antioxidant function. Journal of Biological Chemistry 286: 15085–15094. 10.1074/jbc.M110.210971 21367861 PMC3083210

[43] Allison A (2001) The possible role of vitamin k deficiency in the pathogenesis of alzheimer’s disease and in augmenting brain damage associated with cardiovascular disease. Medical hypotheses 57: 151–155. 10.1054/mehy.2001.1307 11461163

[44] Huy PDQ, Yu YC, Ngo ST, Thao TV, Chen Cp, et al (2013) In silico and in vitro characterization of anti-amyloidogenic activity of vitamin k3 analogues for alzheimer’s disease. Biochimica et Biophysica Acta (BBA)-General Subjects 1830: 2960–2969. 10.1016/j.bbagen.2012.12.026 23295971

[45] Chen Z, Zhong C (2013) Decoding alzheimer’s disease from perturbed cerebral glucose metabolism: implications for diagnostic and therapeutic strategies. Progress in neurobiology 108: 21–43. 10.1016/j.pneurobio.2013.06.004 23850509

[46] Blázquez E, Velázquez E, Hurtado-Carneiro V, Ruiz-Albusac JM (2014) Insulin in the brain: its pathophysiological implications for states related with central insulin resistance, type 2 diabetes and alzheimer’s disease. Frontiers in endocrinology 5 10.3389/fendo.2014.00161 25346723 PMC4191295

[47] Suh JM, Zeve D, McKay R, Seo J, Salo Z, et al (2007) Adipose is a conserved dosage-sensitive antiobesity gene. Cell metabolism 6: 195–207. 10.1016/j.cmet.2007.08.001 17767906 PMC2587167

[48] Huang G, Ge G, Wang D, Gopalakrishnan B, Butz DH, et al (2011) *α*3 (v) collagen is critical for glucose homeostasis in mice due to effects in pancreatic islets and peripheral tissues. The Journal of clinical investigation 121: 769–783. 10.1172/JCI45096 21293061 PMC3026738

[49] Shiba S, Ikeda K, Azuma K, Hasegawa T, Amizuka N, et al (2014) *γ*-glutamyl carboxylase in osteoblasts regulates glucose metabolism in mice. Biochemical and biophysical research communications 453: 350–355. 10.1016/j.bbrc.2014.09.091 25264202

[50] Gil OD, Zanazzi G, Struyk AF, Salzer JL (1998) Neurotrimin mediates bifunctional effects on neurite outgrowth via homophilic and heterophilic interactions. The Journal of neuroscience 18: 9312–9325. 9801370 10.1523/JNEUROSCI.18-22-09312.1998 PMC6792904

[51] Oláh J, Vincze O, Virók D, Simon D, Bozsó Z, et al (2011) Interactions of pathological hallmark proteins: tubulin polymerization promoting protein/p25, beta-amyloid, and alpha-synuclein. The Journal of Biological Chemistry 286: 34088–34100. 10.1074/jbc.M111.243907 21832049 PMC3190826

[52] Pan Y, Wang KS, Aragam N (2011) Ntm and nr3c2 polymorphisms influencing intelligence: family-based association studies. Progress in Neuro-Psychopharmacology and Biological Psychiatry 35: 154–160. 10.1016/j.pnpbp.2010.10.016 21036197

[53] Blacker D, Bertram L, Saunders AJ, Moscarillo TJ, Albert MS, et al (2003) Results of a high-resolution genome screen of 437 alzheimer’s disease families. Human Molecular Genetics 12: 23–32. 10.1093/hmg/ddg007 12490529

[54] Pasterkamp RJ, Giger RJ (2009) Semaphorin function in neural plasticity and disease. Current opinion in neurobiology 19: 263–274. 10.1016/j.conb.2009.06.001 19541473 PMC2730419

[55] Suzuki K, Okuno T, Yamamoto M, Pasterkamp RJ, Takegahara N, et al (2007) Semaphorin 7a initiates t-cell-mediated inflammatory responses through &agr; 1&bgr; 1 integrin. Nature 446: 680–684. 10.1038/nature05652 17377534

[56] Morote-Garcia JC, Napiwotzky D, Köhler D, Rosenberger P (2012) Endothelial semaphorin 7a promotes neutrophil migration during hypoxia. Proceedings of the National Academy of Sciences 109: 14146–14151. 10.1073/pnas.1202165109 PMC3435204 22891341

[57] El Hour M, Moncada-Pazos A, Blacher S, Masset A, Cal S, et al (2010) Higher sensitivity of adamts12-deficient mice to tumor growth and angiogenesis. Oncogene 29: 3025–3032. 10.1038/onc.2010.49 20208563

[58] Moncada-Pazos A, Obaya AJ, Llamazares M, Heljasvaara R, Suárez MF, et al (2012) Adamts-12 metalloprotease is necessary for normal inflammatory response. Journal of Biological Chemistry 287: 39554–39563. 10.1074/jbc.M112.408625 23019333 PMC3501021

[59] Caccamo A, De Pinto V, Messina A, Branca C, Oddo S (2014) Genetic reduction of mammalian target of rapamycin ameliorates alzheimer’s disease-like cognitive and pathological deficits by restoring hippocampal gene expression signature. The Journal of Neuroscience 34: 7988–7998. 10.1523/JNEUROSCI.0777-14.2014 24899720 PMC4044255

[60] Maiese K (2014) Taking aim at alzheimer’s disease through the mammalian target of rapamycin. Annals of medicine 46: 587–596. 10.3109/07853890.2014.941921 25105207 PMC4250432

[61] Tcherkezian J, Cargnello M, Romeo Y, Huttlin EL, Lavoie G, et al (2014) Proteomic analysis of cap-dependent translation identifies larp1 as a key regulator of 5 top mrna translation. Genes & development 28: 357–371. 10.1101/gad.231407.113 24532714 PMC3937514

[62] Crameri A, Biondi E, Kuehnle K, Lütjohann D, Thelen KM, et al (2006) The role of seladin-1/dhcr24 in cholesterol biosynthesis, app processing and a*β* generation in vivo. The EMBO journal 25: 432–443. 10.1038/sj.emboj.7600938 16407971 PMC1383521

[63] Stefani M, Liguri G (2009) Cholesterol in alzheimer’s disease: unresolved questions. Current Alzheimer Research 6: 15–29. 10.2174/156720509787313899 19199871

[64] Sun JH, Yu JT, Tan L (2014) The role of cholesterol metabolism in alzheimer’s disease. Molecular neurobiology: 1–19. 10.1007/s12035-014-8749-y 24838626

[65] Pasterkamp RJ, Peschon JJ, Spriggs MK, Kolodkin AL (2003) Semaphorin 7a promotes axon outgrowth through integrins and mapks. Nature 424: 398–405. 10.1038/nature01790 12879062

[66] Gama L, Wilt SG, Breitwieser GE (2001) Heterodimerization of calcium sensing receptors with metabotropic glutamate receptors in neurons. Journal of Biological Chemistry 276: 39053–39059. 10.1074/jbc.M105662200 11489900

[67] Tharmalingam S, Daulat AM, Antflick JE, Ahmed SM, Nemeth EF, et al (2011) Calcium-sensing receptor modulates cell adhesion and migration via integrins. Journal of Biological Chemistry 286: 40922–40933. 10.1074/jbc.M111.265454 21969374 PMC3220485

[68] Um JW, Kaufman AC, Kostylev M, Heiss JK, Stagi M, et al (2013) Metabotropic glutamate receptor 5 is a coreceptor for alzheimer a*β* oligomer bound to cellular prion protein. Neuron 79: 887–902. 10.1016/j.neuron.2013.06.036 24012003 PMC3768018

[69] Mason BJ (2001) Treatment of alcohol-dependent outpatients with acamprosate: a clinical review. Journal of Clinical Psychiatry 62: 42–48. 11584875

[70] Chumakov I, Nabirotchkin S, Cholet N, Milet A, Boucard A, et al (2015) Combining two repurposed drugs as a promising approach for alzheimer’s disease therapy. Scientific reports 5 10.1038/srep07608 25566747 PMC5378993

[71] Scart-Gres C, Haddad R, Foucquier J, Guedj M, Hugon J, et al (2014) First evidence of pxt00864 effect in the treatment of mild ad: results on 30 patients from the pleodial study. CTAD abstracts, The Journal of Prevention of Alzheimer’s Disease,Volume 1, Number 3, 232–233.

[72] Rubin DB (2004) Multiple imputation for nonresponse in surveys, volume 81 John Wiley & Sons.

[73] Troyanskaya O, Cantor M, Sherlock G, Brown P, Hastie T, et al (2001) Missing value estimation methods for dna microarrays. Bioinformatics 17: 520–525. 10.1093/bioinformatics/17.6.520 11395428

[74] Oba S, Sato Ma, Takemasa I, Monden M, Matsubara Ki, et al (2003) A bayesian missing value estimation method for gene expression profile data. Bioinformatics 19: 2088–2096. 10.1093/bioinformatics/btg287 14594714

[75] Browning SR, Browning BL (2007) Rapid and accurate haplotype phasing and missing-data inference for whole-genome association studies by use of localized haplotype clustering. The American Journal of Human Genetics 81: 1084–1097. 10.1086/521987 17924348 PMC2265661

[76] Howie BN, Donnelly P, Marchini J (2009) A flexible and accurate genotype imputation method for the next generation of genome-wide association studies. PLoS genetics 5: e1000529 10.1371/journal.pgen.1000529 19543373 PMC2689936

[77] Consortium GP, et al (2010) A map of human genome variation from population-scale sequencing. Nature 467: 1061–1073. 10.1038/nature09534 20981092 PMC3042601

[78] Liu JZ, Mcrae AF, Nyholt DR, Medland SE, Wray NR, et al (2010) A versatile gene-based test for genome-wide association studies. The American Journal of Human Genetics 87: 139–145. 10.1016/j.ajhg.2010.06.009 20598278 PMC2896770

[79] Evangelou M, Smyth DJ, Fortune MD, Burren OS, Walker NM, et al (2014) A method for gene-based pathway analysis using genomewide association study summary statistics reveals nine new type 1 diabetes associations. Genetic epidemiology 38: 661–670. 10.1002/gepi.21853 25371288 PMC4258092

[80] Lesnick TG, Papapetropoulos S, Mash DC, Ffrench-Mullen J, Shehadeh L, et al (2007) A genomic pathway approach to a complex disease: axon guidance and parkinson disease. PLoS genetics 3: e98 10.1371/journal.pgen.0030098 17571925 PMC1904362

[81] Eleftherohorinou H, Wright V, Hoggart C, Hartikainen AL, Jarvelin MR, et al (2009) Pathway analysis of gwas provides new insights into genetic susceptibility to 3 inflammatory diseases. PloS one 4: e8068 10.1371/journal.pone.0008068 19956648 PMC2778995

[82] Carbonetto P, Stephens M (2013) Integrated enrichment analysis of variants and pathways in genome-wide association studies indicates central role for il-2 signaling genes in type 1 diabetes, and cytokine signaling genes in crohn’s disease. PLoS genetics 9: e1003770 10.1371/journal.pgen.1003770 24098138 PMC3789883

